# The ATF6-EGF Pathway Mediates the Awakening of Slow-Cycling Chemoresistant Cells and Tumor Recurrence by Stimulating Tumor Angiogenesis

**DOI:** 10.3390/cancers12071772

**Published:** 2020-07-02

**Authors:** Jaebeom Cho, Hye-Young Min, Honglan Pei, Xuan Wei, Jeong Yeon Sim, Shin-Hyung Park, Su Jung Hwang, Hyo-Jong Lee, Sungyoul Hong, Young Kee Shin, Ho-Young Lee

**Affiliations:** 1 Creative Research Initiative Center for concurrent control of emphysema and lung cancer, College of Pharmacy, Seoul National University, Seoul 08826, Korea; gslife@snu.ac.kr (J.C.); snoopy77@snu.ac.kr (H.-Y.M. ); peihonglan@naver.com (H.P.); 15251757369@163.com (X.W.); sjyeon1103@snu.ac.kr (J.Y.S.); 2 College of Pharmacy and Research Institute of Pharmaceutical Sciences, Seoul National University, Seoul 08826, Korea; psyji043@hanmail.net (S.-H.P.); sungyoul@snu.ac.kr (S.H.); ykeeshin@snu.ac.kr (Y.K.S.); 3 Department of Molecular Medicine and Biopharmaceutical Sciences, Graduate School of Convergence Science and Technology and College of Pharmacy, Seoul National University, Seoul 08826, Korea; 4 College of Pharmacy, Inje University, Gimhae, Gyungnam 50834, Korea; sama3575@naver.com (S.J.H.); hjlee@inje.ac.kr (H.-J.L.)

**Keywords:** slow-cycling cancer cells, chemoresistance, angiogenesis, tumor recurrence

## Abstract

Slow-cycling cancer cells (SCCs) with a quiescence-like phenotype are believed to perpetrate cancer relapse and progression. However, the mechanisms that mediate SCC-derived tumor recurrence are poorly understood. Here, we investigated the mechanisms underlying cancer recurrence after chemotherapy, focusing on the interplay between SCCs and the tumor microenvironment. We established a preclinical model of SCCs by exposing non-small-cell lung cancer (NSCLC) cells to either the proliferation-dependent dye carboxyfluorescein diacetate succinimidyl ester (CFSE) or chemotherapeutic drugs. An RNA sequencing analysis revealed that the established SCCs exhibited the upregulation of a group of genes, especially epidermal growth factor (EGF). Increases in the number of vascular endothelial growth factor receptor (VEGFR)-positive vascular endothelial cells and epidermal growth factor receptor (EGFR) activation were found in NSCLC cell line- and patient-derived xenograft tumors that progressed upon chemotherapy. EGFR tyrosine kinase inhibitors effectively suppressed the migration and tube formation of vascular endothelial cells. Furthermore, activating transcription factor 6 (ATF6) induced the upregulation of EGF, and its antagonism effectively suppressed these SCC-mediated events and inhibited tumor recurrence after chemotherapy. These results suggest that the ATF6-EGF signaling axis in SCCs functions to trigger the angiogenesis switch in residual tumors after chemotherapy and is thus a driving force for the switch from SCCs to actively cycling cancer cells, leading to tumor recurrence.

## 1. Introduction

Despite great advances in its diagnosis and treatment, cancer is the leading cause of death worldwide [1]. Although several anticancer therapies—including conventional chemotherapy, targeted therapy, and immunotherapy—have been developed and are used in the clinic [2], many cancer patients eventually develop tumor relapse, the major cause of cancer-related death [3]. Non-small cell lung cancer (NSCLC), which accounts for 80–85% of lung cancer cases [4], is not an exception. At present, chemotherapy is the standard treatment option for patients with NSCLC [5]; however, up to 75–77% of patients with early-stage NSCLC who have undergone surgical resection with or without adjuvant/neoadjuvant chemotherapy ultimately develop relapse [3,6,7]. To improve the prognosis of NSCLC patients, it is important to better understand the biology of cancer recurrence and develop efficacious strategies to suppress disease progression.

Multiple studies have suggested dormancy as the main contributor to cancer relapse and progression [8,9]. Since most chemotherapeutics target highly proliferating cancer cells [8], a small population of dormant cancer cells in minimal residual disease is believed to cause tumor recurrence [10,11]. Quiescence, a non-proliferative or slow proliferative state with reversible growth arrest [12], is a functional standard of cellular dormancy [9]. Upon exposure to environmental cellular stresses, including chemotherapy, cell-intrinsic mechanisms such as p38 mitogen-activated protein kinase (MAPK) activation and the unfolded protein response (UPR) were shown to induce growth arrest and increase the survival capacity of dormant cancer cells [13,14]. The UPR, which is mediated by activating transcription factor 6 α (ATF6α), inositol requiring enzyme 1 alpha (IRE1α), and protein kinase R-like endoplasmic reticulum kinase (PERK) [15], has been implicated in chemoresistance and cancer development [13,16,17]. The activation of p38 caused by cellular stresses was found to protect dormant cancer cells from chemotherapy-induced insults [18]. Additionally, various soluble factors secreted in the tumor microenvironment (TME) modulate extrinsic mechanisms, leading to either the suppression or stimulation of cellular dormancy [8,9,10].

Although the mechanisms underlying the control of cancer cell dormancy have remained largely unknown, one effective therapeutic strategy for preventing tumor relapse/progression may be targeting the mechanisms that convert dormant cancer cells into cancer cells in a proliferative state. One such strategy would be the suppression of angiogenesis-associated factors that ensure an appropriate supply of oxygen and nutrients for tumor outgrowth and metastasis [19]. Indeed, angiogenic burst from the tumor microenvironment is known to cause escape from dormancy [20,21,22,23], and angiogenesis failure in disseminated tumor cells was found to mediate tumor dormancy [9]. Various factors, including growth factors, cytokines, proteases, trace elements, and other endogenous mediators, have been identified as activators or inhibitors of angiogenesis [19]. It remains unclear, however, how the expression of such factors is regulated.

In the present study, we aimed to investigate the mechanism that stimulates the exit of NSCLC cells from a dormant state and to develop novel strategies to target dormant NSCLC cells using slow-cycling/dormant cancer cells (SCCs) derived from NSCLC cell lines and patient-derived xenograft (PDX) tumors. We found that established SCCs exhibited the transcriptional upregulation of epidermal growth factor (EGF) through the activation of the ATF6-UPR branch, which, in turn, stimulated angiogenesis in residual tumors after chemotherapy. The clinical relevance of these findings was validated by using NSCLC cell line-derived tumors and PDX tumors that survived chemotherapy. We further showed that the ablation of ATF6 or inactivation of EGF signaling are promising strategies to break the interplay between dormant cancer cells and their microenvironment, thereby inhibiting the angiogenesis-mediated awakening of dormant cancer cells.

## 2. Results

### 2.1. Increased Vasculature in NSCLC Cell Line- and Patient-Derived Xenograft Tumors that Relapsed Upon Completion of Chemotherapy 

**Figure 1:**
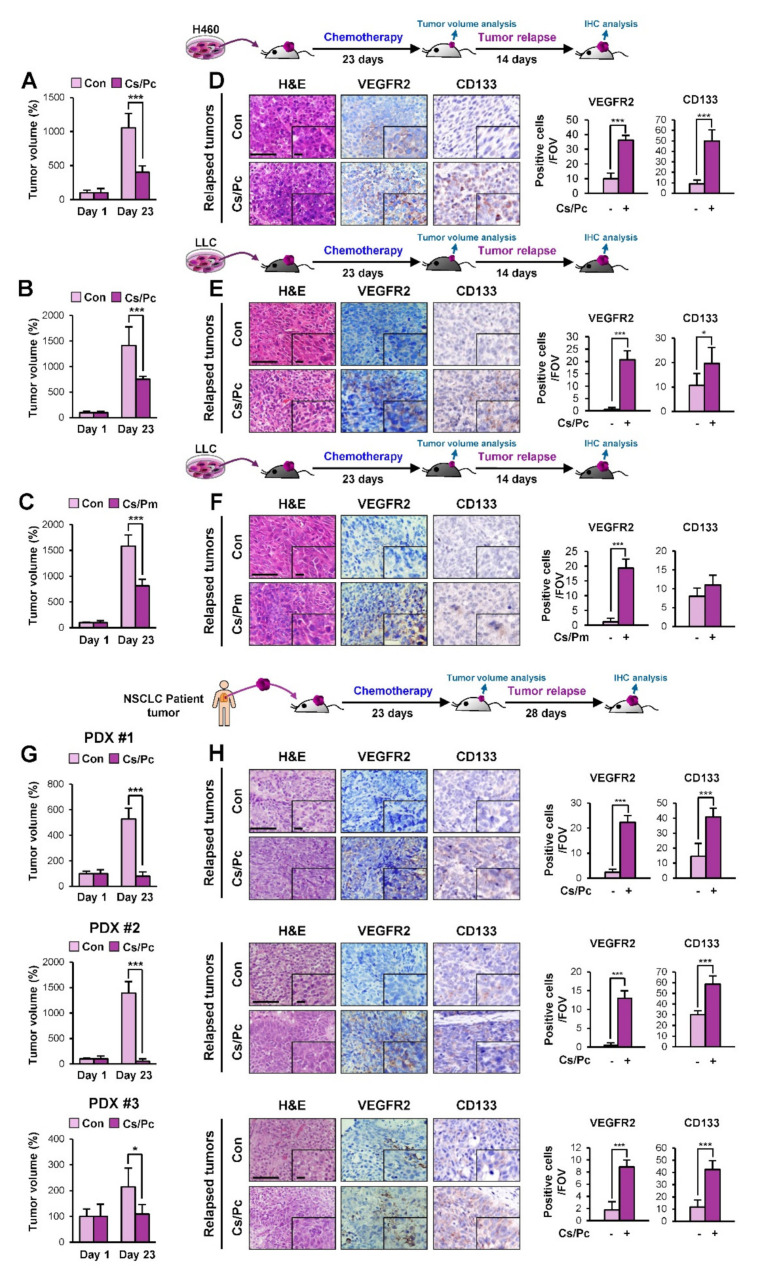
Increased accumulation of vascular endothelial cells in lung tumors derived from those that progressed after chemotherapy in vivo. (**A–C, G**) Changes in tumor growth in mice bearing H460 xenograft tumors (Con: *n* = 4; Cs/Pc: *n* = 5) (**A**), LLC allograft tumors [(**B**) Con: *n* = 8; Cs/Pc: *n* = 12; (**C**) Con: *n* = 8; Cs/Pm: *n* = 9], and lung patient-derived xenograft (PDX) tumors derived from three different non-small-cell lung cancer (NSCLC) patients [PDX #1 (Con: *n* = 8; Cs/Pc: *n* = 4); PDX #2 (Con: *n* = 6; Cs/Pc: *n* = 6); PDX #3 (Con: *n* = 6; Cs/Pc: *n* = 5)] (**G**) subjected to three cycles of combinatorial chemotherapy (each cycle consists of treatment with paclitaxel (Pc; 20 mg/kg) and cisplatin (Cs; 3 mg/kg) in combination for a day or cisplatin (Cs; 3 mg/kg) and pemetrexed (Pm; 50 mg/kg) in combination for a day, followed by a drug holiday for 6 days). (**D–F, H**). Immunohistochemistry (IHC) analyses showing the recruitment of vascular endothelial cells (VEGFR2^+^) and endothelial progenitor cells (CD133^+^) in tumors that progressed after chemotherapy. Quantification of cells positive for each marker per field of view (FOV, *n* = 12 from at least three tumors) is depicted as a graph (**D–F, H**). Scale bar: 50 μm (**D–F, H**). Scale bar (inset): 10 μm (**D–F, H**). For all panels, the bars represent the mean ± SD. *p < 0.05 and ***p < 0.001, as determined by two-tailed Student’s *t*-test.

Given the role of angiogenesis in cancer development and relapse [8,19], we analyzed changes in the vasculature of residual NSCLC xenograft tumors that relapsed upon the completion of chemotherapy. To this end, we exposed immune-compromised nonobese diabetic/severe combined immunodeficiency (NOD/SCID) mice bearing H460 xenograft tumors (Figure 1A) or immune-competent C57BL/6 mice bearing tumor allografts of Lewis lung carcinoma (LLC) cells (Figure 1B,C) to a short-term, clinically relevant combinatorial chemotherapy consisting of either cisplatin and paclitaxel (Cs/Pc) or cisplatin and pemetrexed (Cs/Pm). As shown in Figure 1A–C, these combinatorial chemotherapies induced significant decreases in the growth of tumors. However, the residual tumors recommenced growth after chemotherapy completion, and the relapsed tumors (collected 2 weeks after the completion of chemotherapy) showed significantly increased numbers of vascular endothelial cells (VEGFR2^+^) and endothelial progenitor cells (EPCs; CD133^+^) compared with the control tumors (Figure 1D–F).

To determine the clinical significance of these findings, we further administered the combinatorial chemotherapy to NSCLC PDX tumors. Among a number of PDX tumors established in NOD/SCID mice, three PDX tumors (PDX #1 - PDX #3) were found to be chemosensitive. These PDX tumors rapidly shrank during combinatorial chemotherapy (Figure 1G), but the residual tumors remained and eventually regrew. The relapsed PDX tumor tissues (collected 1 month after the completion of chemotherapy) consistently showed an increased number of vascular endothelial cells and EPCs compared to their vehicle-treated control tumors (Figure 1H). These results supported the potential role of the angiogenic switch in residual tumors for relapse after chemotherapy.

### 2.2. EGF-Related Gene Sets Are Significantly Enriched in NSCLC Cells with Quiescence-Like Phenotypes Derived from the NSCLC Cell Line and PDX Tumors 

**Figure 2:**
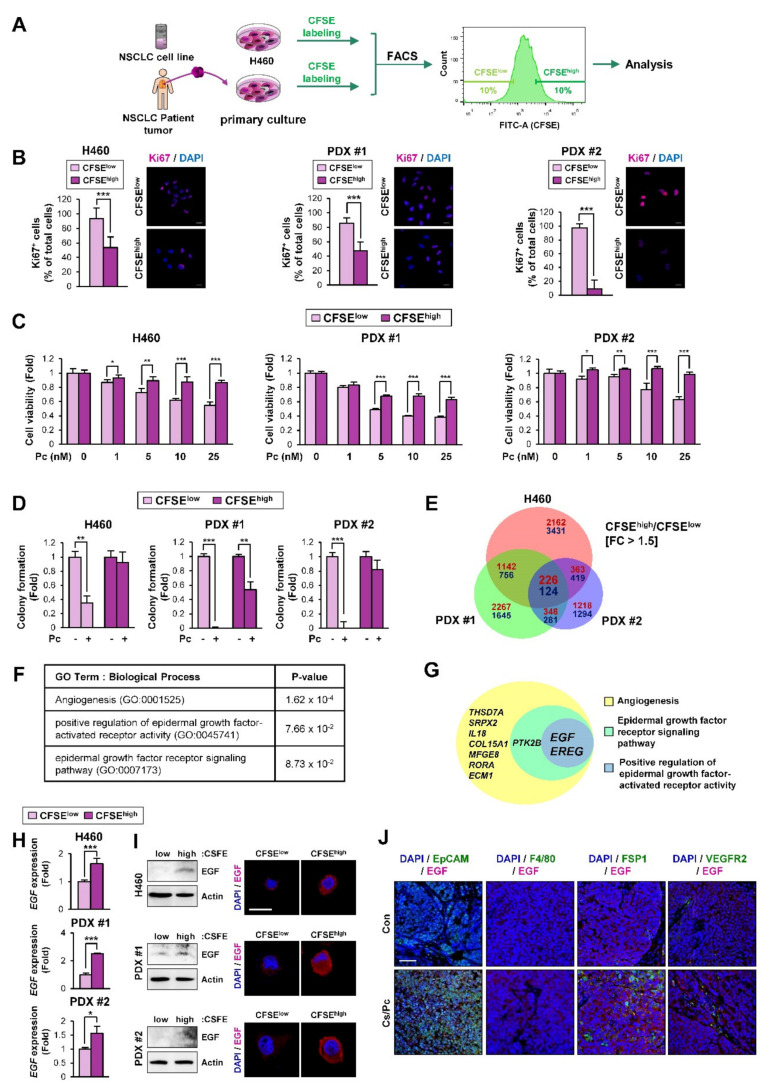
Enrichment of epidermal growth factor (EGF)-associated genes in the slow-cycling carboxyfluorescein diacetate succinimidyl ester (CFSE)^high^ cell population. (**A**) A representative image showing the flow cytometric cell sorting of CFSE^high^ and CFSE^low^ cell populations. The top 10% and the bottom 10% of total cells were defined as CSFE^high^ and CSFE^low^ populations, respectively. (**B**) Reduced cell proliferation in the CFSE^high^ population was determined by immunofluorescence staining using an anti-Ki67 antibody. Scale bar: 20 μm. (**C, D**) Decreased sensitivity to paclitaxel in the CFSE^high^ population was determined by the 3-(4,5-dimethylthiazol-2-yl)-2,5-diphenyltetrazolium bromide (MTT) (**C**) and anchorage-dependent colony formation (**D**) assays. (**E**) A Venn diagram showing commonly regulated genes in the CFSE^high^ population from H460 cells and PDXs compared with those from the corresponding CFSE^low^ populations. The Venn diagram was drawn using the freely available web-based tool [30] (**F**) Enrichment of GO terms associated with angiogenesis and the EGF pathway in the CFSE^high^ population from H460 cells and PDXs, as determined by DAVID analysis. (**G**) A Venn diagram showing commonly regulated genes in the following GO terms: angiogenesis, epidermal growth factor receptor signaling pathway, and positive regulation of epidermal growth factor-activated receptor activity. The freely available web-based tool [30] was used for drawing the Venn diagram. (**H**) Commonly upregulated *EGF* expression in the CFSE^high^ population compared with the corresponding CFSE^low^ population was determined by real-time PCR. (**I**) Increase in the EGF protein expression in the CFSE^high^ population compared with the CFSE^low^ population was determined by Western blot and immunofluorescence (IF) analyses. Scale bar: 20 μm. (**J**) Elevation of EGF expression in EpCAM^+^ tumor cells in H460 xenograft tumors that relapsed after the completion of combinatorial chemotherapy (Cs/Pc) was determined by double IF analysis. Scale bar: 50 μm. For all panels, the bars represent the mean ± SD. *p < 0.05, **p < 0.01, and ***p < 0.001, as determined by two-tailed Student’s *t*-test.

Accumulating evidence has shown the role of SCCs in cancer relapse [9]. Since SCCs have been observed in actively proliferating cancer cell lines and tumors [24,25,26], we endeavored to obtain SCCs by adapting the use of the proliferation-dependent dye carboxyfluorescein diacetate succinimidyl ester (CFSE) [24,26]. To this end, the H460 NSCLC cell line and a primary culture of PDX tumor cells were labeled with CFSE and separated into CFSE^high^ and CFSE^low^ populations by flow cytometry (Figure 2A). Compared with the CFSE^low^ population, the CFSE^high^ population displayed a slower growth and greater chemoresistance, as demonstrated by decreased Ki67 positivity (Figure 2B) but increased viability (Figure 2C) and colony-forming capacity in the presence of paclitaxel (Figure 2D). These findings suggest that the CFSE^high^ population is a distinct NSCLC SCC subpopulation. We then performed an RNA-sequencing analysis to identify differentially expressed gene signatures in the CFSE^high^ versus the CFSE^low^ populations. We found that 350 genes were commonly modulated in the CFSE^high^ populations derived from H460 cells and PDXs compared with their corresponding CFSE^low^ populations (upregulated: 226 genes; downregulated: 124 genes) (Figure 2E). A gene ontology (GO) analysis using DAVID (database for annotation, visualization, and integrated discovery) bioinformatics resources [27] revealed that 37 GO terms associated with biological processes were enriched in the common upregulated gene set (Table S1). Among them, the GO terms associated with angiogenesis and epidermal growth factor (EGF) signaling were significantly enriched in the common upregulated gene set (Figure 2F). Based on the role of EGF signaling in angiogenesis [28], correlations between the genes involved in these GO terms (angiogenesis, the epidermal growth factor receptor signaling pathway, and the positive regulation of epidermal growth factor-activated receptor activity) were analyzed. As shown in Figure 2G, two genes—*EREG* (encoding epiregulin) and *EGF* (encoding EGF)—were commonly enriched in these terms. These genes belong to the EGF family [29], confirming the association of EGF with angiogenesis. We validated the expression of these two genes in H460 cell- and PDX-derived CFSE^high^ and CFSE^low^ populations. As shown in Figure 2H and Figure S1, the *EGF* expression was commonly upregulated in the CFSE^high^ populations compared with the corresponding CFSE^low^ populations, whereas the *EREG* expression was not consistently modulated in the CFSE^high^ populations. We also confirmed the elevation of EGF protein expression in the CFSE^high^ populations compared with the corresponding CFSE^low^ populations by Western blot and immunofluorescence (IF) analyses (Figure 2I). Moreover, double IF analyses using antibodies against EGF and cell type-specific markers (EpCAM for tumor cells, F4/80 for macrophages, FSP1 for fibroblasts, and VEGFR2 for endothelial cells) in relapsed H460 xenograft tumors upon the completion of combinatorial chemotherapy confirmed the upregulation of EGF in EpCAM^+^ tumor cells (Figure 2J). According to these results, we chose EGF for further investigation. These results suggest that the EGF and EGF-associated gene sets are likely involved in the biological and functional features of SCCs.

### 2.3. Activation of EGFR in Residual NSCLC Cell Line-Derived and PDX Tumors after Chemotherapy

**Figure 3:**
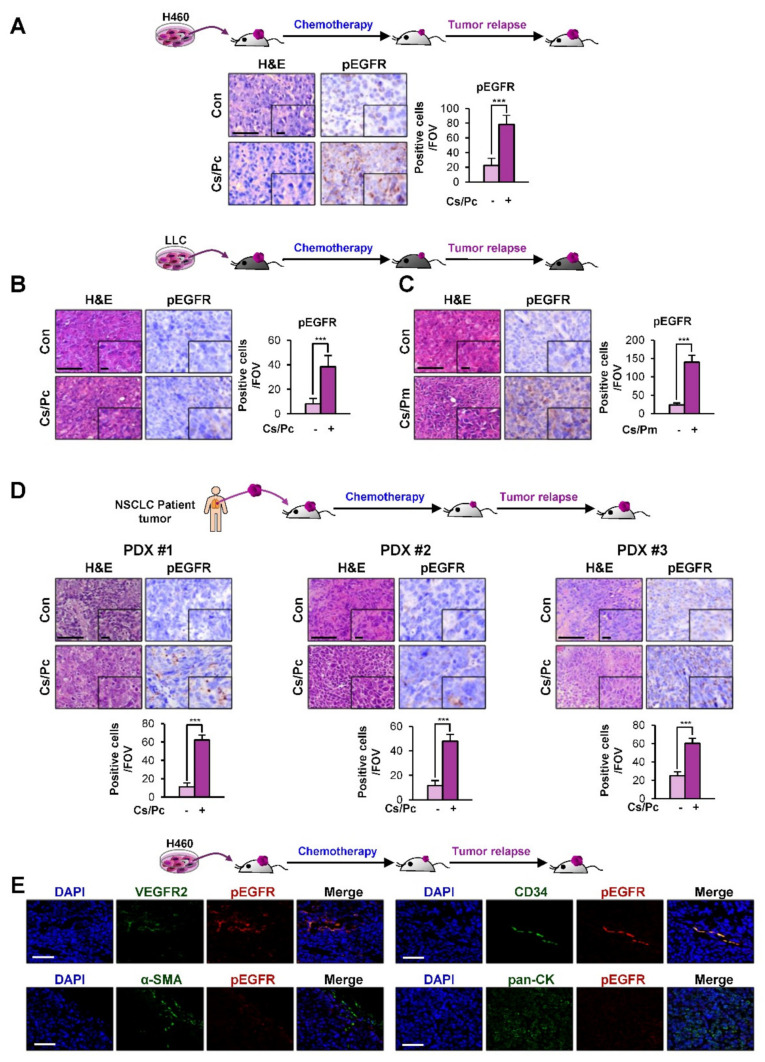
Increased expression of phosphorylated epidermal growth factor receptor (EGFR) in tumors derived from those that relapsed after chemotherapy in vivo. (**A–D**) IHC analyses to assess the activation of EGFR [phosphorylated EGFR (pEGFR; phosphorylation at Y1068-positive)] in H460 xenograft tumors (**A**), Lewis lung carcinoma (LLC) allograft tumors (**B, C**), and PDX tumors (**D**) that relapsed after chemotherapy. Quantification of cells positive for each marker per field of view (FOV, *n* = 12 from at least three tumors) is depicted as a graph. (**E)** Elevation of pEGFR expression in VEGFR2^+^ endothelial cells or CD34^+^ EPCs in H460 xenograft tumors that relapsed after chemotherapy (Cs/Pc) was determined by a double IF analysis. Scale bar: 50 μm (**A–E**). Scale bar (inset): 10 μm (**A–D**). For all panels, the bars represent the mean ± SD. ***p < 0.001, as determined by two-tailed Student’s *t*-test.

We next performed an immunohistochemistry (IHC) analysis of the residual H460 xenograft tumors, LLC allograft tumors, and PDX tumors after chemotherapy (Figure 1). We observed significant increases in the activated (phosphorylated) EGFR (pEGFR) (Y1068) levels in all of the residual xenograft tumors after chemotherapy compared to their vehicle-treated control tumors (Figure 3A–D). We further examined the cell types expressing pEGFR in tumors relapsed after chemotherapy. To this end, we carried out double IF staining using antibodies against pEGFR and each cell type-specific marker [VEGFR2 for endothelial cells, CD34 for EPCs, α-smooth muscle actin (α-SMA) for fibroblasts, and pan-cytokeratin (pan-CK) for tumor cells] in H460 xenograft tumors that relapsed after the Cs/Pc combinatorial treatment. We found that pEGFR was mainly expressed in the CD34^+^ EPCs and VEGFR2^+^ endothelial cells, indicating the activation of the EGFR signaling in endothelial cells (Figure 3E). Together, these findings suggest the stimulation of tumor angiogenesis through the transcriptional upregulation of EGF expression in SCCs and the subsequent activation of the EGF-mediated signaling pathways in endothelial cells.

### 2.4. EGF Expression Was Significantly Upregulated in the H460 NSCLC Cell Subpopulation Carrying Quiescence-Like Phenotypes

**Figure 4:**
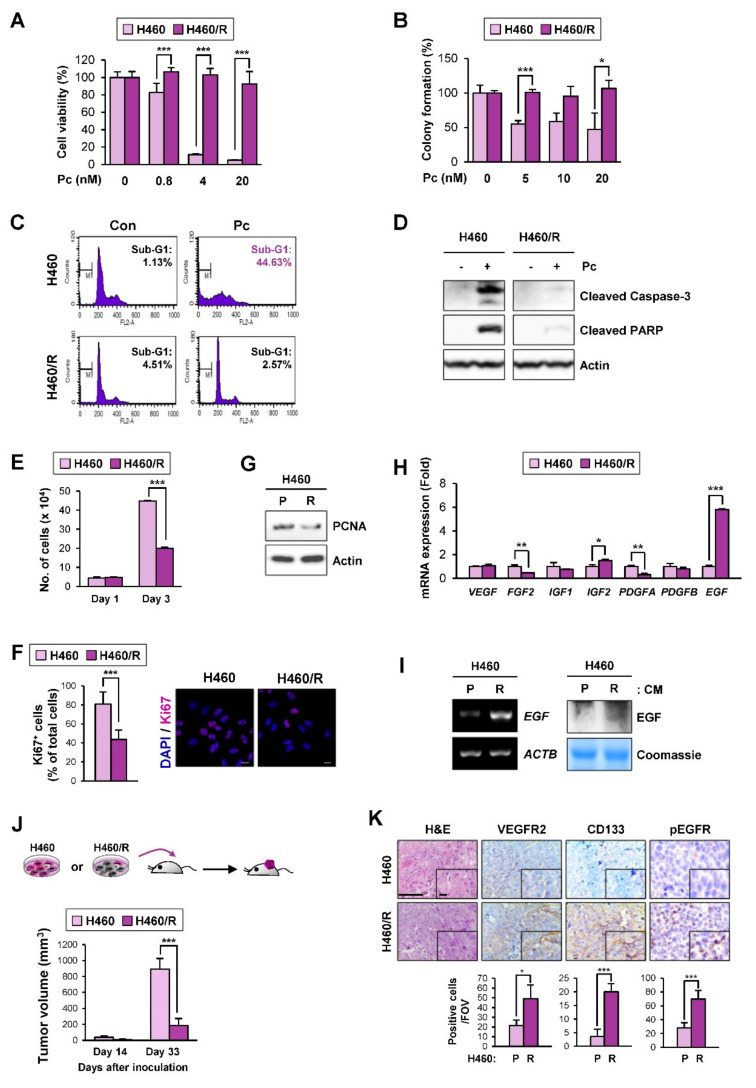
Increased EGF expression in the quiescent-like chemoresistant cell population. (**A–D**) Acquisition of resistance to paclitaxel in H460/R cells compared with the corresponding parental cells (H460) was determined by a crystal violet assay (**A**), a soft agar colony formation assay (**B**), a flow cytometric analysis of PI-stained cells (**C**), and a Western blot analysis determining the cleavage of poly (ADP-ribose) polymerase (PARP) and caspase-3 (**D**). (**E–G**) Reduced cell proliferation in H460/R cells compared with H460 cells was determined by a cell counting assay (**E**), an analysis of Ki67-positive cells using immunofluorescence staining (scale bar: 20 μm) (**F**), and a Western blot analysis to determine the protein expression of proliferating cell nuclear antigen (PCNA) (**G**). (**H**) The mRNA expression of several proangiogenic soluble factors in H460/R cells compared with H460 cells was determined by a real-time PCR. (**I**) Left: *EGF* mRNA expression in H460 and H460/R cells was determined by RT-PCR. Right: EGF protein levels in conditioned medium (CM) derived from H460 and H460/R cells were determined by Western blot analysis. (**J**) Changes in tumor volumes in xenograft tumors derived from H460 (*n* = 4) and H460/R (*n* = 4) cells at days 14 and 33 after cell inoculation. (**K**) IHC analyses to assess the accumulation of vascular endothelial cells (VEGFR2^+^) and EPCs (CD133^+^) and the activation of EGFR [pEGFR (Y1068)^+^] in H460/R tumor xenografts. Scale bar: 50 μm. Scale bar (inset): 10 μm. Bottom: quantification of cells positive for each marker per field of view (FOV, *n* = 12 from at least three tumors) is depicted as a graph. For all panels, the bars represent the mean ± SD. *p < 0.05, **p < 0.01, and ***p < 0.001, as determined by two-tailed Student’s *t*-test.

Conventional chemotherapy is widely used for various types of human cancer but is ineffective against dormant cancer cells [31]. Hence, we attempted to enrich a chemoresistant NSCLC cell subpopulation with a quiescence-like phenotype by exposing H460 cells to paclitaxel (Pc), a chemotherapeutic agent used for the treatment of lung cancer [32]. During the Pc treatment, most of the cells died, but small surviving subpopulations eventually produced colonies after 4 months of selection. Unlike its parental cancer cells, the established chemoresistant subline (H460/R) exhibited minimal changes in viability (Figure 4A), colony-forming ability (Figure 4B), and apoptotic activity (Figure 4C,D) in the presence of Pc. Notably, the H460/R cells appeared to be in a state of slow cycling, as evidenced by the significant decreases in the number of cells (Figure 4E); the Ki67 positivity (Figure 4F); and the expression of proliferating cell nuclear antigen (PCNA), a marker of proliferating cells [33] (Figure 4G). More importantly, the analysis of several proangiogenic factors by real-time PCR and RT-PCR analyses confirmed the significant upregulation of *EGF* mRNA expression in H460/R cells compared with H460 cells (Figure 4H,I). We also confirmed that the EGF protein expression was greater in conditioned medium (CM) derived from H460/R cells than in CM derived from H460 cells (Figure 4I).

We next compared the tumorigenic capacity of H460 and H460/R cells in NOD/SCID mice. The H460/R cells successfully developed xenograft tumors in mice, with a markedly delayed tumor onset compared with their parental H460 cells (Figure 4J). These findings suggested that the interaction with the tumor microenvironment stimulated the awakening of SCCs in vivo. We further found a significant increase in VEGFR2 and CD133 staining in H460/R-derived xenograft tumors compared with H460-derived xenograft tumors (Figure 4K). More importantly, xenograft tumors derived from the H460/R cells showed a significant increase in the phosphorylated EGFR (pEGFR) (Y1068) levels compared to those derived from the H460 cells (Figure 4K). These findings suggest the potential of SCCs to stimulate angiogenesis through the transcriptional upregulation of *EGF* expression. Hence, we used the H460/R subpopulation as an in vitro model in which to study the biology of dormant cancer cells.

### 2.5. SCC-Derived EGF Stimulates Migration and Vascular Endothelial Cell Tube Formation

**Figure 5:**
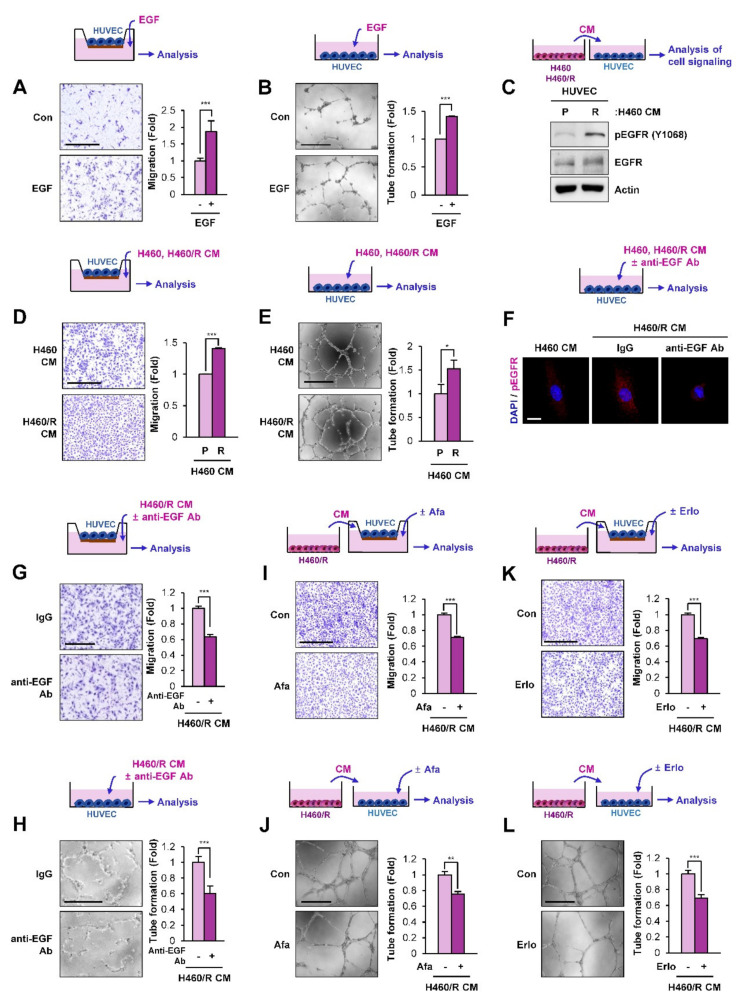
Association of EGF with proangiogenic behavioral changes in human umbilical vein endothelial cells (HUVECs) mediated by communication between H460/R cells and HUVECs. (**A, B**) The increased migration (**A**) and tube formation (**B**) of HUVECs treated with EGF (50 ng/mL) were determined by migration and tube formation assays, respectively. Scale bar: 500 μm. (**C)** The activation of EGFR in HUVECs treated with conditioned medium (CM) derived from H460/R cells compared with those treated with CM derived from H460 cells was determined by a Western blot analysis. (**D, E**) The increased migration **(D**) and tube formation (**E**) of HUVECs treated with CM derived from H460/R cells by comparison with those treated with CM derived from H460 cells were determined by migration and tube formation assays, respectively. Scale bar: 500 μm. (**F–H**) Inhibition of H460/R CM-induced EGFR phosphorylation **(F**), migration (**G**), and tube formation (**H**) by co-incubation with an anti-EGF neutralizing antibody (1 μg/mL) in HUVECs. Scale bars: 20 μm (**F**), 500 μm (**G, H)** (**I, K**) Blockade of the H460/R CM-induced migration of HUVECs by treatment with afatinib (Afa; 20 nM) (**I**) and erlotinib (Erlo; 5 μM) (**K**). Scale bar: 500 μm. (**J, L**) Inhibition of H460/R CM-induced tube formation in HUVECs by treatment with afatinib (Afa; 20 nM) (**J**) and erlotinib (Erlo; 5 μM) (**L**). Scale bar: 500 μm. For all panels, the bars represent the mean ± SD. *p < 0.05, **p < 0.01, and ***p < 0.001, as determined by two-tailed Student’s *t*-test.

Because EGF plays an important role in regulating angiogenesis [28], we hypothesized that EGF secreted from SCCs plays an important role in angiogenesis. Indeed, treatment with EGF significantly induced the migration (Figure 5A) and tube formation (Figure 5B) of human umbilical vein endothelial cells (HUVECs). We next determined the effects of CM from H460 or H460/R cells on HUVECs. We found that treatment with CM derived from H460/R cells induced increases in EGFR phosphorylation (Figure 5C), migration (Figure 5D), and tube-forming capacity (Figure 5E) in HUVECs. Moreover, when EGF in H460/R-derived CM was inactivated by co-incubation with an anti-EGF neutralizing antibody, the EGFR phosphorylation (Figure 5F), migration (Figure 5G), and tube-forming capacity (Figure 5H) in HUVECs were significantly inhibited. We further analyzed the effects of H460/R cell-derived CM on HUVECs, in which EGFR signaling is inactivated by treatment with EGFR tyrosine kinase inhibitors, such as afatinib [34] and erlotinib [35]. We found that treatment of HUVECs with afatinib (Figure 5I,J) and erlotinib (Figure 5K,L) significantly suppressed their migration (Figure 5I,K) and tube formation (Figure 5J,L) stimulated by treatment with H460/R cell-derived CM. These results indicated the capacity of EGF secreted from chemoresistant SCCs to chemoattract vascular endothelial cells and stimulate their angiogenic activities.

### 2.6. ATF6 Mediates the Angiogenesis-Stimulating Activities of SCCs by the Transcriptional Upregulation of EGF

**Figure 6:**
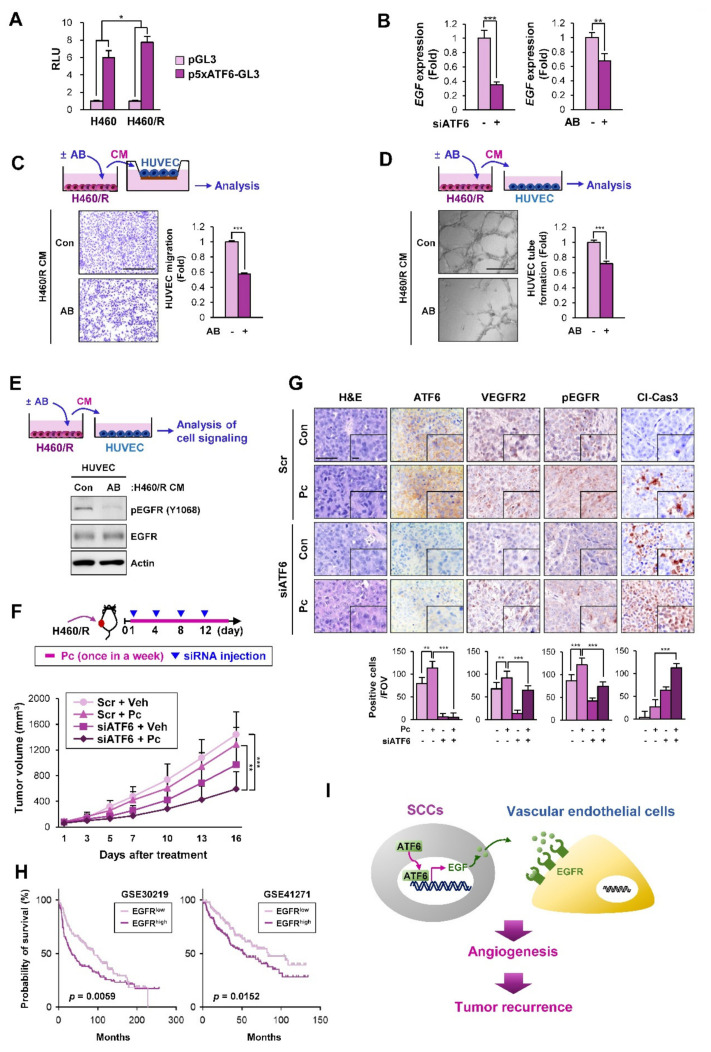
Involvement of ATF6 in angiogenesis mediated by communication between H460/R cells and HUVECs. (**A**) Upregulated ATF6-mediated transcriptional activity in H460/R cells compared with H460 cells was determined with a luciferase reporter assay using a reporter vector containing five repeats of the ATF6 DNA-binding site (p5xATF6-GL3). (**B**) Downregulated *EGF* expression in H460/R cells transfected with ATF6 small interfering RNA (siRNA) or the ATF6 inhibitor ABESF (AB; 150 μM) compared with that in cells transfected with scrambled siRNA (Scr) or cells treated with vehicle (DMSO), respectively, was determined by real-time PCR. (**C, D**) Abrogation of migration (**C**) and tube formation (**D**) in HUVECs exposed to CMs derived from H460/R cells treated with 4-(2-Aminoethyl)benzenesulfonyl fluoride hydrochloride (ABESF) (AB; 150 μM). Scale bar: 500 μm. (**E**) Attenuation of EGFR phosphorylation in HUVECs treated with CM derived from H460/R cells treated with ABESF (AB; 150 μM) was determined by a Western blot analysis. (**F**) Attenuated tumor growth and enhanced sensitivity to paclitaxel (Pc) treatment caused by the intratumoral injection of liposome-encapsulated ATF6 siRNA (Scr + Veh and Scr + Pc: *n* = 7; siATF6 + Veh and siATF6 + Pc: *n* = 6). (**G**) IHC analyses showing decreases in vascular endothelial cell accumulation and EGFR activation and the upregulation of cleaved caspase-3 (Cl-Cas3) in tumors derived from mice treated with ATF6 siRNA in combination with Pc. Quantification of cells positive for each marker shown as the number of positive cells per field of view (FOV, *n* = 12 from at least three tumors) is depicted as a graph. Scale bar: 50 μm. Scale bar (inset): 10 μm. (**H**) Kaplan–Meier survival plots showing the association of *EGFR* expression with the poor overall survival of patients with NSCLC were determined by the analysis of two datasets (GSE30219 and GSE41271) available in the Gene Expression Omnibus (GEO) database. (**I**) Proposed model for the recurrence of chemoresistant cells by EGF-mediated tumor angiogenesis. For all panels, the bars represent the mean ± SD. *p < 0.05, **p < 0.01, and ***p < 0.001, as determined by two-tailed Student’s *t*-test (**A**, **B**, **C**, **D**) or one-way ANOVA with Tukey’s post-hoc test (**F**, **G**).

We investigated the mechanism underlying the transcriptional upregulation of *EGF* in H460/R cells. We recently found that NSCLC SCCs exhibited the transcriptional upregulation of various soluble factors through the activation of ATF6 [36]. For this reason, we determined whether ATF6 was involved in the expression of *EGF* in H460/R cells and their angiogenesis-stimulating activities. A reporter gene assay using a luciferase vector carrying five repeats of ATF6-binding motifs [37] revealed elevated luciferase reporter activity in H460/R cells compared with parental H460 cells (Figure 6A). We further found that both ATF6 inactivation through treatment with the ATF6 inhibitor 4-(2-Aminoethyl)benzenesulfonyl fluoride hydrochloride (AEBSF) [38] and the small interfering RNA (siRNA)-mediated silencing of ATF6 expression significantly suppressed the *EGF* mRNA expression in H460/R cells (Figure 6B), indicating the involvement of ATF6 in the transcription of *EGF*. HUVECs exposed to H460/R cell-derived CM in the presence of AEBSF showed a significantly decreased migration (Figure 6C) and tube formation (Figure 6D) compared to those incubated with H460/R cell-derived CM alone. We further confirmed that EGFR phosphorylation in HUVECs, which was stimulated by treatment with H460/R-derived CM, was markedly attenuated by treatment with ABESF (Figure 6E). 

We next determined the effects of ATF6 depletion, both alone and in combination with chemotherapy, on the growth of H460/R xenograft tumors, the number of vascular endothelial cells, and the phosphorylation of EGFR in the tumors (Figure 6F, top). Treatment with liposome-encapsulated ATF6 siRNA effectively suppressed the growth of H460/R xenograft tumors (Figure 6F, bottom). The ATF6 siRNA-mediated antitumor effects were further enhanced upon the addition of Pc treatment. We also confirmed that the siRNA-mediated loss of ATF6 expression induced an obvious decrease in the number of VEGFR^+^ vascular endothelial cells and the tumor expression of pEGFR (Figure 6G). Moreover, the Pc-mediated expression of cleaved caspase-3 (Cl-Cas3) was significantly upregulated by treatment with ATF6 siRNAs, confirming the enhancement of the antitumor effect of Pc by the siRNA-mediated silencing of the ATF6 expression (Figure 6G). An analysis of a publicly available database also revealed that NSCLC patients carrying a high *EGFR* expression showed a poor overall survival, indicating the prognostic impact of EGFR expression in NSCLC (Figure 6H). These results suggest that ATF6-mediated EGF expression in SCCs plays an important role in angiogenesis-stimulating activities, resulting in a switch from SCCs to actively cycling cancer cells and eventually leading to tumor recurrence. Several mechanisms, including CAFs and hypoxia, have been shown to contribute to both chemoresistance and angiogenesis [39,40,41]. In fact, the role of fibroblasts recruited to residual tumors in tumor recurrence has been demonstrated in our recent publication [36]. We also observed the upregulation of HIF-1α expression, a marker of tumor hypoxia [42], in relapsed tumors after chemotherapy (Figure S2). These findings collectively suggest that various mechanisms may contribute to both chemoresistance and tumor relapse.

## 3. Discussion

In the present study, we demonstrated a mechanism by which SCCs in residual tumors after chemotherapy induce tumor recurrence. Using an in vitro model of SCCs to understand the biology of cellular dormancy, we identified that dormant NSCLC cells secrete EGF in the TME through the increased activity of the ATF6 transcription factor. EGF acted on vascular endothelial cells in a paracrine manner as a proangiogenic factor to stimulate their migration and morphogenesis for vascular formation, leading to the outgrowth of SCCs to initiate tumor recurrence (Figure 6I). Additional studies further demonstrated that ATF6 ablation using liposome-encapsulated ATF6 siRNA reduced angiogenesis-stimulating activities in SCCs and thus suppressed tumor recurrence. Collectively, these results suggest that ATF6-mediated EGF expression in SCCs is a target with which to block tumor recurrence.

Despite extensive efforts to develop effective anticancer strategies for the treatment of NSCLC, the five-year survival of NSCLC patients is still approximately 20%, due mainly to the diagnosis of this disease at advanced stages [43]. Most patients who have undergone surgical resection, even those with early-stage NSCLC, eventually develop recurrent tumors at distant sites [6,7]. Dormant cancer cells in residual tumors after anticancer therapy are believed to be crucial for relapsed tumor formation [9]. Several mechanisms underlying tumor dormancy, such as cellular, angiogenic, and immunogenic dormancy, which reflect the intrinsic kinetics of cancer cell growth and the proliferation-death balance in tumors mediated through their interaction with the neighboring environment, have been proposed [8]. Among these mechanisms, the role of dormant cancer cells in chemoresistance and tumor relapse/progression has been emphasized [13]. However, the precise inter- and intracellular networks underlying cellular dormancy and their role in tumor recurrence are largely unknown, mainly due to the lack of experimental model systems for dormant cancer cells for repetitive works.

Based on previous reports that a small subpopulation of SCCs that cause chemoresistance and tumor recurrence [44] were present in even rapidly growing tumors [25] and cancer cell lines [24], we recently established an in vitro model of SCCs (H460/R, H1299/R) by exposing H460 and H1299 cells to paclitaxel (Pc), cisplatin (Cs), or pemetrexed (Pm) [36]. These SCCs appeared to proliferate slowly and survive chemotherapy in vitro but generated tumors in mice. Hence, we hypothesized that these SCCs could induce specific changes within the microenvironment to initiate tumor recurrence. Indeed, SCCs secrete various proinflammatory cytokines, which chemoattract fibroblasts to the TME, leading to collagen deposition and PGE_2_ production in residual tumors after chemotherapy [36]. The subsequent activation of integrin/Src- and EP receptor-mediated signaling pathways stimulate the outgrowth of SCCs to initiate tumor recurrence [36]. Here, we extended our study to discover further molecular changes in SCCs. We attempted to isolate SCCs from H460 cell lines by adapting the use of the proliferation-dependent dye CFSE, which distinguishes subpopulations of slowly and rapidly growing cells, in addition to utilizing a recently established in vitro model of SCCs. Our RNA-seq analysis revealed that the SCCs exhibited transcriptional regulation in several genes encoding EGF, EGF-related proteins, and angiogenesis. Despite the arrested growth and reduced metabolic activity of quiescent cells [45], several genes associated with cell cycle regulation, cell-cell communication, and metabolism are transcriptionally modulated in quiescent cells to protect them from terminal differentiation and apoptosis and thus maintain their survival [46]. Supporting this notion, the observed genetic changes may support the activation of signal transduction for the maintenance and survival of SCCs, eventually leading dormant cancer cells to awaken and resume proliferation in vivo.

The interaction between tumor cells and surrounding stromal cells has been shown to regulate the maintenance and awakening of dormant cancer cells [47]. Notably, established SCCs exhibited the increased transcription of *EGF*, a well-known and typical growth factor involved in the growth and survival of tumor cells [48]. EGF is also known to induce VEGFR expression and vascular endothelial growth factor (VEGF) and basic fibroblast growth factor (bFGF) production via the activation of the EGFR-signaling pathway [28,49,50]. Since increased vasculature may increase the nutrient and oxygen supply, facilitating the outgrowth of dormant/quiescent cells, and EGF is involved in tumor angiogenesis by acting on vascular endothelial cells in both an indirect and a direct manner [28], we reasoned that EGF produced by SCCs acts as a proangiogenic factor that contributes to the awakening of dormant cells in vivo. In support of this notion, EGF released from dormant cells appeared to chemoattract vascular endothelial cells and stimulate their vascularization. Furthermore, residual H460 xenograft tumors, LLC allograft tumors, and PDX tumors that survived chemotherapy consistently displayed a markedly increased number of VEGFR2^+^ vascular endothelial cells along with increased EGFR activation compared to their corresponding control tumors.

Our subsequent mechanistic studies revealed that ATF6, a component of three UPR signaling branches, acted as a transcriptional factor to induce *EGF* expression in the established SCCs. The ER is crucial for maintaining cellular homeostasis by regulating the intracellular calcium concentration and the synthesis and correct folding of proteins [51,52]. Homeostatic perturbation under hazardous circumstances, including chemotherapy, hypoxia, and starvation, causes misfolded proteins to accumulate in the ER [15]. This condition, named ER stress, evokes the UPR through mediating the dissociation of the ER chaperone BiP/GRP78 from three UPR sensor proteins—PERK, ATF6, and IRE1α—and activating these sensors [53]. These UPR sensors rapidly increase the transcription of several target genes involved in restoring ER homeostasis [53]. The dysregulation of the UPR has been implicated in several pathological conditions, such as cancer development [51] and anticancer drug resistance [13]. In particular, the ATF6-signaling branch has been shown to support the survival of dormant cells by activating the Rheb/mTOR pathway [14]. The ATF6 pathway is also involved in chemoresistance [53,54] and cytokine production under ER stress [55]. These previous findings and our current data suggest that sustained ER stress caused by environmental stress may activate the ATF6 branch of the UPR and induce the release of various soluble factors, especially EGF, which in turn promote the survival of dormant cells and trigger angiogenic switching in vascular endothelial cells, eventually leading to tumor recurrence. Therefore, targeting ATF6 can be considered a novel approach to prevent tumor recurrence. In support of this notion, pharmacological or genomic approaches targeting ATF6 induced a significant decrease in EGF transcription and angiogenesis-stimulating activities in SCCs, effectively suppressing the SCC-derived tumor growth. Although additional studies are required to evaluate the effectiveness of this ATF6-targeting strategy on tumor angiogenesis and tumor recurrence in relevant preclinical models, these results collectively suggest the potential of the ATF6-mediated transcriptional control of EGF expression as a target to prevent recurrent tumor formation. 

Recent studies have suggested that cells rapidly activate the UPR in response to hormones or growth factors, including estrogen and EGF, to expand the protein folding capacity and endure anticipated ER stress caused by protein secretion (e.g., the secretion of massive amounts of immunoglobulins in B cells during the differentiation into plasma cells) or hormone/growth factor-induced cell proliferation [56,57]. This phenomenon, termed anticipatory UPR activation, is cytoprotective and confers resistance to various stresses such as chemotherapy. In the present study, we demonstrate that EGF released from residual tumors induces the activation of EGFR in vascular endothelial cells and stimulates vessel formation, eventually leading to angiogenesis and tumor relapse. However, the secreted EGF could have amplified the EGF-dependent anticipatory UPR in residual tumor cells. Taken together, the positive feedback loop between EGF/EGFR and UPR may cooperate for the survival of cancer cells in hazardous microenvironments and the stimulation of tumor angiogenesis, ultimately resulting in tumor relapse.

In summary, the present study shows the mechanism how surviving cancer cells in residual tumors after chemotherapy cause tumor relapse. We showed the changes in residual tumors (NSCLC cell line- or patient-derived tumor xenografts in immune-compromised NOD/SCID mice or LLC allografts in immune-competent C57BL/6 mice) after chemotherapy at a pre-defined time point and demonstrated that the ATF6-mediated production of EGF in SCCs is one of the mechanisms responsible for tumor relapse. We further confirmed that the regulation of the ATF6-mediated stimulation of the EGF pathway significantly enhanced the chemosensitivity of these tumors and delayed tumor relapse. However, these experimental models have limitations in recapitulating actual clinical contexts, wherein a surgical resection of the tumor mass post chemotherapy followed by the assessment of the tumor relapse was not possible. Further studies utilizing clinically relevant experimental models that reflect diverse local and systemic factors involved in the process of tumor relapse are warranted to better understand the biology of cancer dormancy. Additional preclinical and clinical studies are also required to evaluate the effectiveness of targeting the ATF6-EGF-signaling cascade in suppressing tumor recurrence. 

## 4. Materials and Methods

### 4.1. Cell Culture

The human NSCLC line (H460 cells) and a murine lung cancer cell line (LLC cells) were purchased from the American Type Culture Collection (ATCC; Manassas, VA, USA). Human umbilical vein endothelial cells (HUVECs) were purchased from Thermo Fisher Scientific, Waltham, MA, USA). The cells were cultured in RPMI 1640 medium (H460 cells) or DMEM (for LLC cells) supplemented with 10% fetal bovine serum (FBS) and antibiotics (all from WelGENE, Kyeongsan-si, Republic of Korea) and maintained at 37°C with 5% CO_2_ in a humidified atmosphere. The HUVECs were cultured in endothelial cell basal medium [EBM-2 (Lonza Inc., Allendale, NJ, USA)] supplemented with EGM-2 SingleQuots (Lonza). HUVECs between passages 3 and 8 were used. Chemotherapy-resistant cells were generated by continuous exposure to increasing concentrations of corresponding chemotherapeutic drugs for more than 6 months. NSCLC cells and their chemotherapy-resistant subpopulations were authenticated and validated using the AmpFISTR Identifiler PCR Amplification Kit (Applied Biosystems, Foster, CA; cat. No. 4322288) in 2013, 2016, and 2017. Cells passaged for fewer than 6 months after receipt and resuscitated validated cells were used in this study.

### 4.2. Reagents

Antibodies against cleaved caspase 3, ATF6, pEGFR (Y1068), EGFR, CD133, and VEGFR2 were purchased from Cell Signaling Technology (Danvers, MA, USA). Antibodies against actin, VEGFR2, CD34 were purchased from Santa Cruz Biotechnology (Dallas, TX, USA). Antibodies against PCNA, CD133, FSP1 (S100A4), EpCAM, pan-cytokeratin (pan-CK), and Ki67 were purchased from Abcam (Cambridge, UK). A mouse monoclonal anti-cleaved PARP antibody was purchased from BD Biosciences (San Jose, CA, USA). Anti-EGF antibody was purchased from GeneTex (Irvine, CA, USA). An antibody against murine F4/80 was purchased from Bio-Rad Laboratories (Hercules, CA, USA). Anti-α-smooth muscle actin (α-SMA) antibody, crystal violet, propidium iodide (PI), and other chemicals were purchased from Sigma-Aldrich (St. Louis, MO, USA) unless otherwise specified.

### 4.3. Cell Viability Assay

Cells (2 ×10^3^ cells/well in 96-well plates or 2 ×10^4^ cells/well in 24-well plates) were incubated for different time intervals or treated with various concentrations of test compounds for three days. The cells were harvested and counted using a hemocytometer or further incubated with the 3-(4,5-dimethylthiazol-2-yl)-2,5-diphenyltetrazolium bromide (MTT solution (final 500 μg/mL) for 2-4 h at 37°C for the MTT assay or fixed with 100% methanol for 30 min at room temperature for the crystal violet assay. For the MTT assay, after the removal of the culture media including the MTT solution, the plates were air-dried and formzan crystals were dissolved in 100% DMSO. The absorbance was measured at 570 nm. For the crystal violet assay, the fixed cells were stained with 0.01% crystal violet for 30 min at room temperature. The stained cells were washed with tap water several times, air-dried, and then dissolved in 100% methanol. The absorbance was measured at 570 nm. The data are presented as percentages compared to the control group.

### 4.4. Anchorage-Dependent Colony Formation Assay

The cells were seeded onto 6-well plates at a density of 300 cells/well. The cells were then allowed to form colonies or treated with test compounds for 10–14 days. The colonies were fixed with 100% methanol, stained with 0.01% crystal violet solution at room temperature, and then washed with deionized water 3–5 times. The colonies were photographed and counted using the ImageJ software (National Institutes of Health, Bethesda, Maryland, USA) [58].

### 4.5. Anchorage-Independent Colony Formation Assay

A total of 0.5 ml of cell suspension (1 × 10^3^ cells/well) mixed with a sterile 1% low-melting agar solution (final concentration of 0.4%) was layered onto a base of 1% solidified low-melting agar in 24-well plates. After the addition of complete medium with or without test materials to the solidified top agar, the cells were incubated for 2 weeks at 37 °C with 5% CO_2_. After incubation, the colonies were stained with an MTT solution, photographed, and counted using the ImageJ software.

### 4.6. Cell Cycle Analysis

The cells were treated with paclitaxel (20 nM) for two days. Adherent and floating cells were collected and fixed in ice-cold 100% methanol overnight at −20 °C. The cells were stained with a PI (50 μg/mL) solution containing RNase A (50 μg/mL) for 30 minutes at room temperature. Apoptotic cells were analyzed by flow cytometry using a FACSCalibur^®^ flow cytometer (BD Biosciences).

### 4.7. Migration Assay

HUVECs were added to the upper chamber of a Transwell insert (6.5-mm insert; Corning, Inc., Corning, NY, USA) coated with gelatin and incubated for 18–19 h. The conditioned medium obtained from the cultured cells treated with or without inhibitors was used as a chemoattractant. The cells were fixed with methanol and stained with hematoxylin. The cells on the bottom of the membrane were counted under a microscope. In total, 12 fields of the membrane were used for counting.

### 4.8. Tube Formation Assay

HUVECs were seeded into CellBIND Surface 6-well plates and grown in EBM-2 media containing CM from the cultured cells treated with or without inhibitors (EBM-2:CM = 1:1). The morphological changes in the HUVECs were photographed, and a three-branch point event was scored as one tube.

### 4.9. Transfection

To knock down the ATF6 expression, the cells were transiently transfected with small interfering RNAs (siRNAs) targeting ATF6 (Bioneer Corp., Daejeon, Republic of Korea) using the JetPRIME transfection reagent (Polyplus-transfection SA, Illkirch, France) according to the manufacturer’s instructions. Scrambled siRNAs (Bioneer) were used as a negative control.

### 4.10. Western Blot Analysis

The cells were lysed using a modified radioimmunoprecipitation assay (RIPA) buffer [50 mM Tris-HCl (pH 7.4), 150 mM NaCl, 1 mM EDTA, 0.25% sodium deoxycholate, 1% Triton X-100] containing various protease and phosphatase inhibitors (100 mM NaF, 5 mM Na_3_VO_4_, 1 mM PMSF, 1 μg/mL aprotinin, 1 μg/mL leupeptin, and 1 μg/mL pepstatin). Cell lysates containing equal amounts (10–30 μg) of protein were resolved using 8% or 15% SDS-PAGE, and the proteins were then transferred onto a PVDF membrane. The membranes were blocked with blocking buffer [3% BSA in TBS containing 0.1% Tween-20 (TBST)] for 1 h at room temperature and then incubated with primary antibodies diluted in a blocking buffer (1:1000) overnight at 4 °C. The membranes were washed with TBST for 1 h at room temperature and then incubated with the corresponding secondary antibodies diluted in 3% skim milk in TBST (1:5000) for 1–2 h at room temperature. The membranes were washed several times with TBST for 1 h, and then the blots were visualized using an enhanced chemiluminescence (ECL) detection kit (Thermo Fisher Scientific). The uncropped Western blots figures can be found in Figure S3.

### 4.11. RT-PCR and Real-Time PCR

The total RNA was isolated using a phenol-chloroform extraction method, reverse transcribed, and analyzed by PCR. The primer sequences used for the PCR analyses are listed in Table 1. The PCR was performed using 2× MyTaq Red Mix (Bioline, London, UK) and gene-specific primers. The following RT-PCR conditions were applied: an initial denaturation step at 94 °C for 5 min; 28–35 cycles of 94 °C for 30 sec, 55–60 °C for 30 sec, and 72 °C for 30 S; and a final elongation step at 72 °C for 5–7 min. The PCR products were separated by 2% agarose gel electrophoresis and visualized using a Gel Doc EZ System (Bio-Rad). For the real-time PCR analysis, we used a SYBR Green-based TOP real™ qPCR 2X PreMIX master mix solution (Enzynomics Co., Ltd., Daejeon, Republic of Korea) and gene-specific primers. The following thermocycler conditions for real-time PCR were applied: preincubation at 95 °C for 5 min; 40 cycles of 95 °C for 15 S 60 °C for 15 sec, and 72 °C for 30 S; and a final melt curve analysis to determine the reaction specificity. The relative quantification of mRNA expression was performed using the comparative CT (cycle threshold) method, as described previously [59].

### 4.12. Luciferase Reporter Assay

To measure the ATF6 transcriptional activity, the cells were transiently cotransfected with p5xATF6-GL3 (a gift from Ron Prywes (Addgene plasmid # 11976; http://n2t.net/addgene:11976; RRID:Addgene_11976)) or empty vector (pGL3) along with pSV-β-Gal using the JetPRIME transfection reagent (Polyplus-transfection SA) according to the manufacturer’s instructions. The cells were harvested with Lysis-Juice (PJK GmbH, Kleinblittersdorf, Germany). The luciferase activity was monitored with Beetle-Juice (PJK GmbH) using a microplate luminometer (Berthold Technologies GmbH & Co. KG, Germany). The β-galactosidase activity was measured using β-Gal-Juice (PJK GmbH) and served as a control to normalize the transfection efficiency.

### 4.13. Animal Studies

All the animal procedures were performed using protocols approved by the Seoul National University Institutional Animal Care and Use Committee. For the xenograft experiments, the cells were subcutaneously injected into the right flanks of 6-week-old NOD/SCID or C57BL/6 mice. For the PDX experiments, the tumors that had been passaged less than 5 times in mice were homogenized into 2 mm^3^ pieces and subcutaneously implanted into the NOD/SCID mice. After the tumor volume reached 50–150 mm^3^, the mice were randomly grouped and treated with either the vehicle or test materials. The tumor growth was determined by measuring the short and long diameters of the tumor with a caliper, and the body weight was measured twice per week to monitor the toxicity. The tumor volume was calculated using the following formula: tumor volume (mm^3^) = (short diameter)^2^ × (long diameter) × 0.5.

### 4.14. IHC Analysis

Sections of the formalin-fixed and paraffin-embedded tissue specimens were deparaffinized, rehydrated, and treated with methanol containing 0.3% hydrogen peroxide. The slides were incubated with primary antibodies overnight at 4 °C, followed by incubation with a biotinylated secondary antibody (Vector Laboratories) for 1 h. Solutions A and B (ABC Elite, Vector Laboratories) were added simultaneously and incubated for 30 min, and the signals were detected using a 3,3^′^-diaminobenzidine (DAB) substrate kit (Vector Laboratories). The slides were further counterstained with hematoxylin.

### 4.15. Immunofluorescence

The cells seeded onto coverslips were fixed with 4% paraformaldehyde for 30 min at room temperature. For staining of formalin-fixed paraffin-embedded (FFPE) tumor tissues, sections (thickness: 4 μm) of FFPE tissues were deparaffinized, rehydrated, and then subjected to antigen retrieval (citrate buffer, pH 6.0). The fixed cells or antigen-retrieved FFPE tissues were incubated with blocking buffer (5% BSA in TBST) and then incubated with anti-Ki67 antibody diluted in TBS containing 1% BSA. After incubation with the corresponding rhodamine-conjugated secondary antibodies, the coverslips were mounted with a mounting solution (Dako, Agilent) containing DAPI. The fluorescence was observed under a fluorescence microscope.

### 4.16. RNA-Sequencing Analysis

A library was constructed using the SENSE mRNA-Seq Library Prep Kit (Lexogen, Vienna, Austria) according to the manufacturer’s recommendations. High-throughput sequencing was then performed as paired-end 100 sequencing using a HiSeq 2000 system (Illumina, San Diego CA, USA). The RNA-Seq reads were mapped using TopHat software to obtain the alignment file. The alignment file was used to assemble the transcripts, estimate the abundance, and detect the differential expression of genes/isoforms using Cufflinks. Library preparation and RNA sequencing were performed as NGS services provided by Ebiogen, Inc. (Seoul, Korea). Parts of the RNA-seq results were deposited in the GEO database (accession No: GSE141218). 

### 4.17. In Silico Analysis

We used publicly available datasets [GSE30219 [60] and GSE41271 [61]] deposited in the GEO database (National Center for Biotechnology Information) to analyze the involvement of *EGFR* expression with the survival of patients with NSCLC. Raw data comprising the gene expression levels and clinical information for each patient sample were manually downloaded and analyzed using the GraphPad Prism 8 (GraphPad Software Inc., San Diego, CA, USA). The probes used to obtain the gene expression values in each dataset were 1565483_at (GSE30219) and ILMN_1728858 (GSE41271). The EGFR^high^ and EGFR^low^ groups were defined based on the median value of the data in each dataset. The log-rank test was used to determine the significance. 

### 4.18. Statistical Analyses

The data are presented as the mean ± SD. All the in vitro experiments were independently performed at least twice, and a representative result is shown. Statistical significance was determined by a two-tailed Student’s *t*-test or one-way analysis of variance (ANOVA) using Microsoft Excel 2010 (Microsoft Corp., Redmond, WA, USA) or GraphPad Prism 8. *p* values less than 0.05 indicated significance.

## 5. Conclusions

The present study demonstrates that the ATF6-mediated production of EGF in SCCs induces the recruitment of vascular endothelial cells and triggers tumor angiogenesis via the activation of the EGFR signaling in vascular endothelial cells. This angiogenesis switch in residual tumors after chemotherapy leads SCCs to awaken and resume proliferation in vivo, eventually resulting in tumor recurrence. These results suggest that targeting the ATF6-EGF signaling cascade would be an efficacious strategy to suppress tumor recurrence. 

## Figures and Tables

**Table 1:** Primer sequences used in this study.

Gene	Forward Sequence (5^‘^-3^‘^)	Reverse Sequence (5^‘^-3^‘^)	Application
*EGF*	CAGACCCTTGAAGGGGGTGT	ATCTGTCTCCAGGCATTGAG	Real-time PCR
*EGF*	AAGAATGGGGGTCAACCAGT	TGAAGTTGGTTGCATTGACC	Real-time PCR
*VEGF*	CCTGGTGGACATCTTCCAGGAGTACC	GAAGCTCATCTCTCCTATGTGCTGGC	Real-time PCR
*FGF2*	TGTGCTAACCGTTACCTGGC	CGTTTCAGTGCCACATACCAA	Real-time PCR
*IGF1*	TGGTGGATGCTCTTCAGTTC	GACAGAGCGAGCTGACTTG	Real-time PCR
*IGF2*	CCGTGCTTCCGGACAACTT	CTGCTTCCAGGTGTCATATTGC	Real-time PCR
*PDGFA*	TTGGCCACCTTGACGCT	CCTGCCCATTCGGAGGAA	Real-time PCR
*PDGFB*	TTTCTCACCTGGACAGGTCG	GAAGGAGCCTGGGTTCCCT	Real-time PCR
*EGF*	GGGAGCCTGAGCAGAAACTT	CTTCAGTGTATGGGCAA	RT-PCR
*ATF6*	ATGAAGTTGTGTCAGAGAACC	CTCTTTAGCAGAAAATCCTAG	Real-time PCR
*HMBS*	CATGTCTGGTAACGGCAATG	GTACGAGGCTTTCAATGTTG	Real-time PCR
*ACTB*	TCATTCCAAATATGAGATGCGTTG	TAGAGAGAAGTGGGGTGGCT	Real-time PCR

## Supplementary Materials

The following are available online at https://www.mdpi.com/2072-6694/12/7/1772/s1: Figure S1: The levels of *EREG* expression in the CFSE^high^ population compared with the CFSEl^ow^ population. *EREG* mRNA expression was determined by real-time PCR. Figure S2: Upregulation of HIF-1α expression in tumors derived from those relapsed after chemotherapy. IHC analyses to examine the levels of HIF-1α expression in H460 xenograft tumors, LLC allograft tumors, and PDX tumors that relapsed after chemotherapy. Quantification of cells positive for each marker per field of view (FOV, *n* = 12 from at least three tumors) is depicted as a graph. Scale bar: 50 μm. Scale bar (inset): 10 μm. The bars represent the mean ± SD. *** *p* < 0.001, as determined by two-tailed Student’s t-test. Figure S3: Uncropped blots with molecular weight markers and densitometry of each band. Table S1: GO Terms (biological process) enriched in the common upregulated gene set, determined by a functional analysis using DAVID.
