# Supplementary material for: The ATF6-EGF Pathway Mediates the Awakening of Slow-Cycling Chemoresistant Cells and Tumor Recurrence by Stimulating Tumor Angiogenesis
Source: Cancers (Basel). 2020 Jul 2;12(7):1772. doi: 10.3390/cancers12071772 (PMC7407555; doi:10.3390/cancers12071772)
Supplement: Supplementary file 1 [file cancers-12-01772-s001.zip › supplementary/cancers-838909-supplementary-done.pdf]

cancers

Supplementary materials

The ATF6-EGF pathway mediates the awakening of slow-cycling chemoresistant cells and tumor recurrence by stimulating tumor angiogenesis

Jaebeom Cho, Hye-Young Min, Honglan Pei, Xuan Wei, Jeong Yeon Sim, Shin-Hyung Park, Su Jung Hwang, Hyo-Jong Lee, Sungyoul Hong, Young Kee Shin and Ho-Young Lee

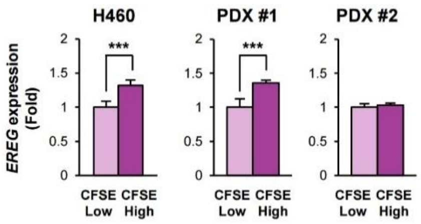

Figure S1. The levels of *EREG* expression in the CFSE^{high} population compared with the CFSE^{low} population. *EREG* mRNA expression was determined by real-time PCR.

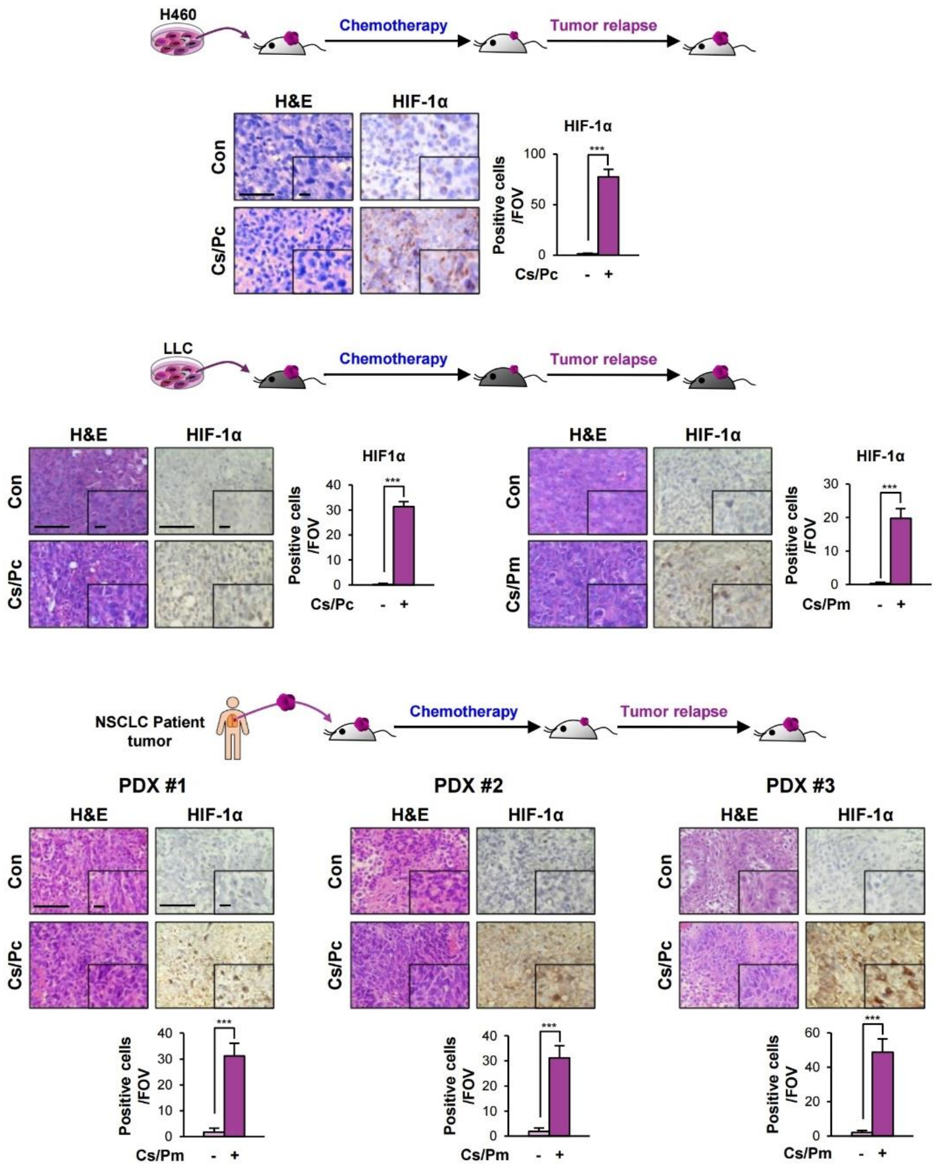

Figure S2. Upregulation of HIF-1α expression in tumors derived from those relapsed after chemotherapy. IHC analyses to examine the levels of HIF-1α expression in H460 xenograft tumors, LLC allograft tumors, and PDX tumors that relapsed after chemotherapy. Quantification of cells positive for each marker per field of view (FOV, n = 12 from at least three tumors) is depicted as a graph. Scale bar: 50 μm (**A-E**). Scale bar (inset): 10 μm. The bars represent the mean ± SD. ***P < 0.001, as determined by two-tailed Student's t-test.

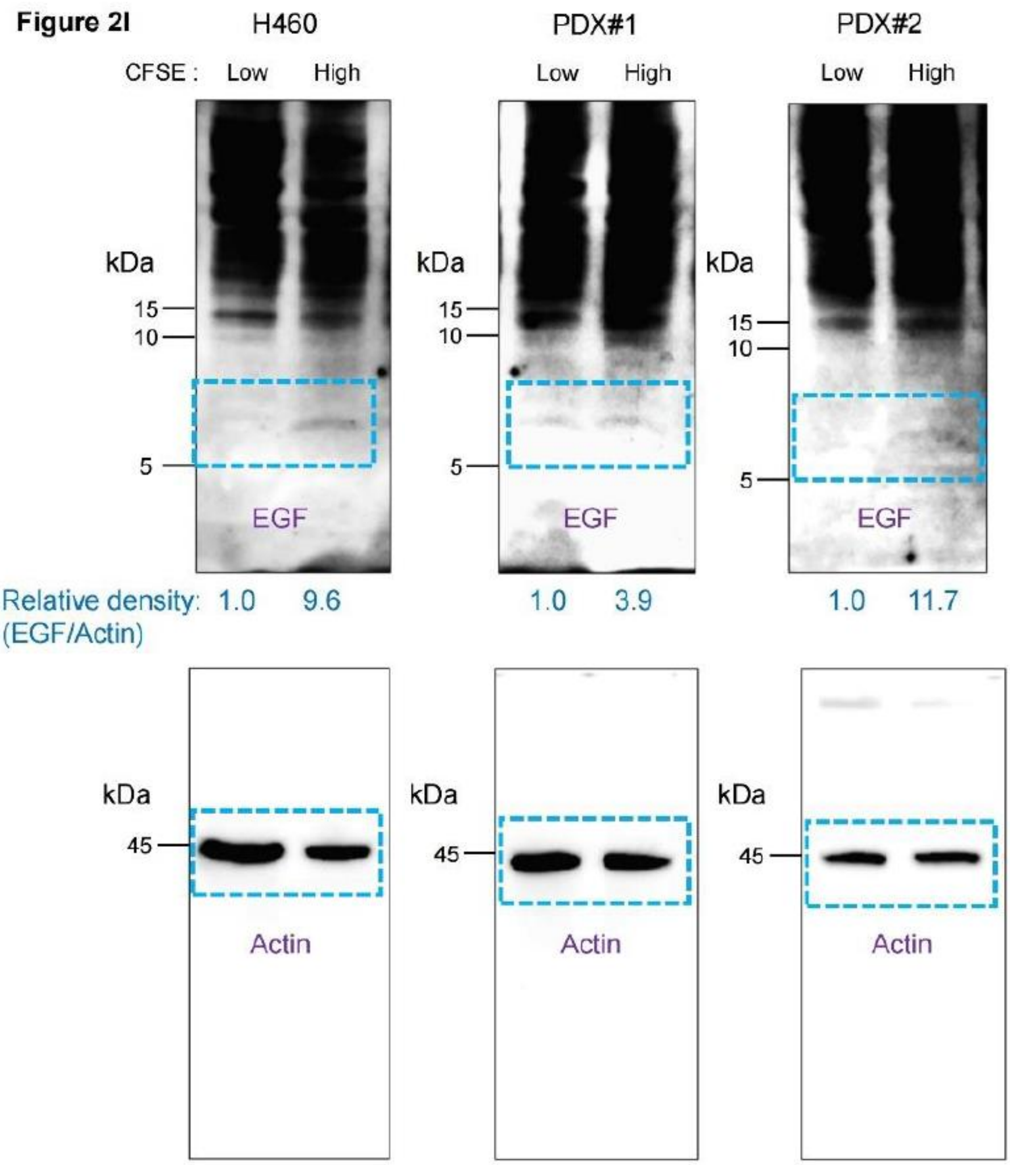
Figure 2I
H460
PDX#1
PDX#2
CFSE : Low High
Low High
Low High
kDa
15
10
5
EGF
Relative density: 1.0 9.6
(EGF/Actin)
1.0 3.9
1.0 11.7
45
Actin

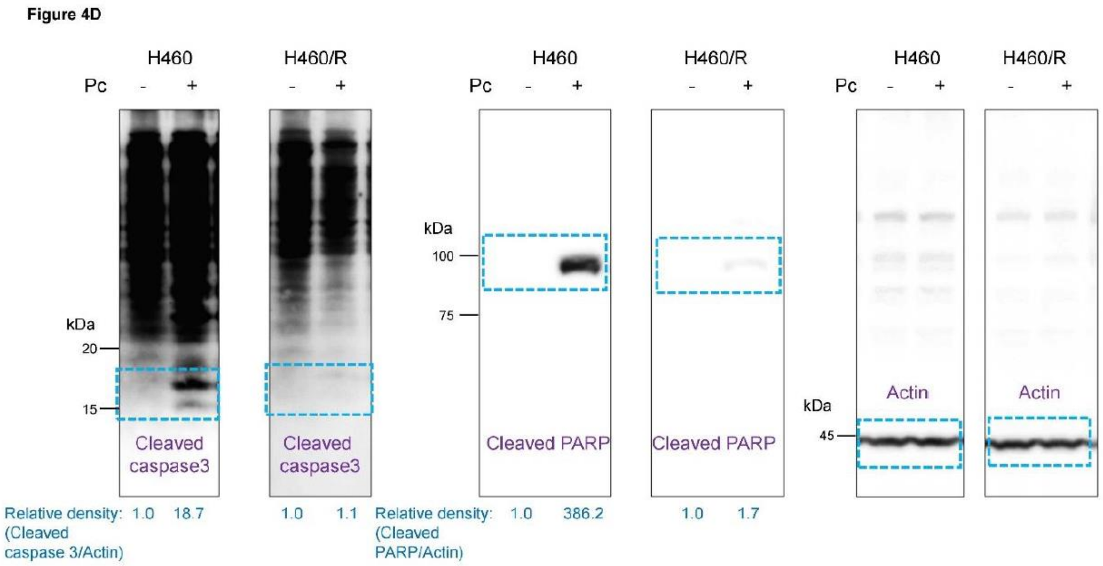
Figure 4D
H460
H460/R
Pc - +
kDa
20
15
100
75
45
Cleaved caspase3
Cleaved PARP
Actin
Relative density: 1.0 18.7
(Cleaved caspase 3/Actin)
1.0 1.1
Relative density: 1.0 386.2
(Cleaved PARP/Actin)
1.0 1.7

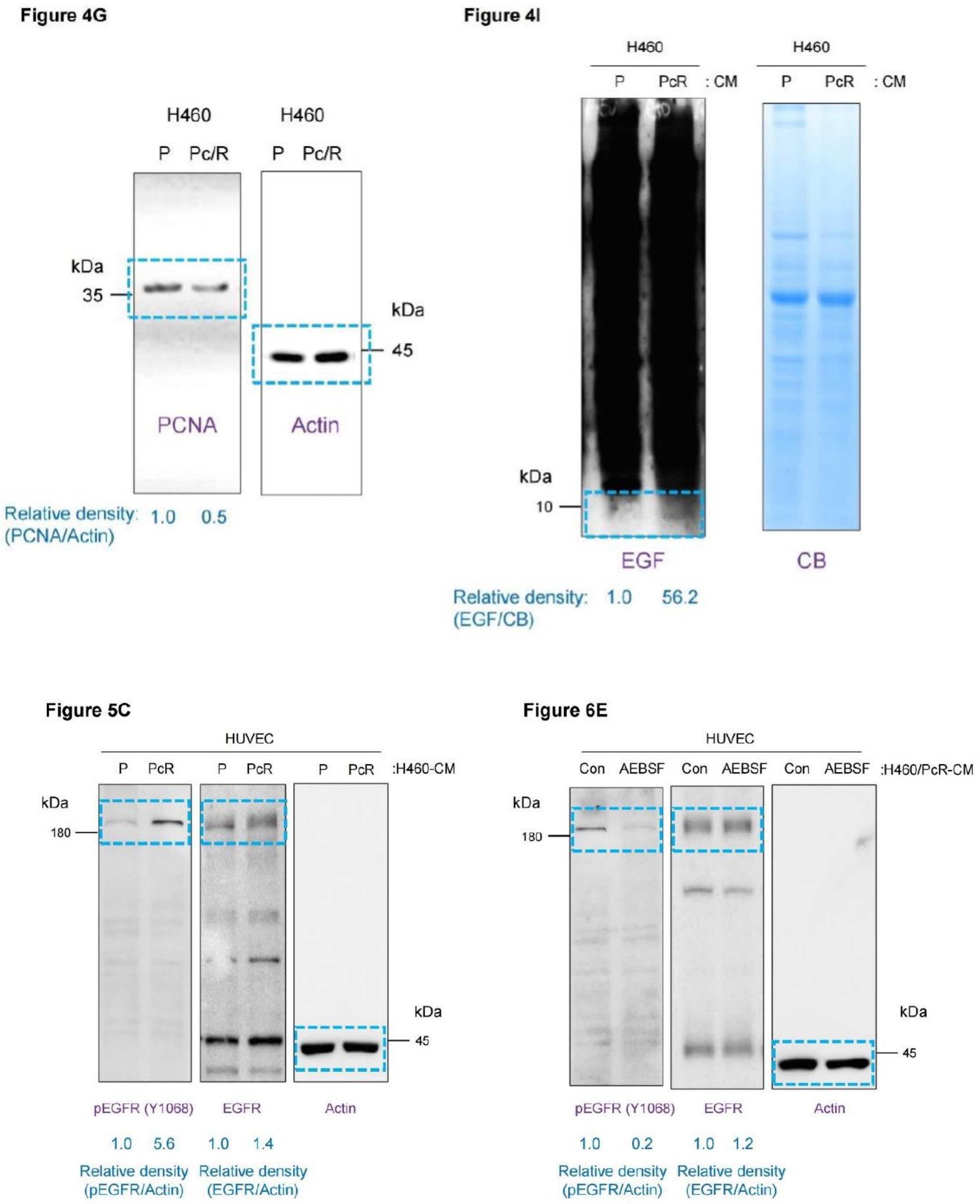

Figure S3. Uncropped blots with molecular weight markers and densitometry of each band.
